# Phenotype and Function of CD25-Expressing B Lymphocytes Isolated from Human Umbilical Cord Blood

**DOI:** 10.1155/2011/481948

**Published:** 2011-09-18

**Authors:** Sylvie Amu, Mikael Brisslert

**Affiliations:** ^1^Department of Rheumatology and Inflammation Research, Sahlgrenska Academy at Göteborg University, P.O. Box 480, 405 30 Göteborg, Sweden; ^2^Institute of Molecular Medicine, Trinity Centre, St. James Hospital, Dublin 8, Ireland

## Abstract

*Background*. We have shown that approximately 30% of human peripheral blood B-cells express CD25. B cells expressing CD25 display a mature phenotype belonging to the memory B-cell population and have a better proliferative and antigen-presenting capacity. The aim of the present study was to characterize the CD25-expressing subset of B cells in human cord blood. *Material and Methods*. Mononuclear cell fraction from human cord blood (*n* = 34) and peripheral adult blood (*n* = 22) was sorted into CD20^+^CD25^+^ and CD20^+^CD25^−^ B-cell populations. Phenotype and function of these B-cell populations were compared using flow cytometry, proliferation, cytokine production, and immunoglobulin secretion. *Results*. CD25-expressing B cells are a limited population of cord blood mononuclear cells representing 5% of the CD20^+^ B cells. They are characterised by high expression of CD5 in cord blood and CD27 in adult blood. CD25-expressing B cells express a functional IL-2 receptor and high levels of CC-chemokine receptors and spontaneously produce antibodies of IgG and IgM subclass. *Conclusions*. CD25 expression is a common denominator of a specific immunomodulatory B-cell subset ready to proliferate upon IL-2 stimulation, possibly ready to migrate and home into the peripheral tissue for further differentiation/action.

## 1. Introduction

B cells are important players of the immune system due to immunoglobulin production and ability to present antigens and to secrete various cytokines. Using different surface marker expression, the B-cell population can be divided into distinct subsets that are of importance during different immunological events (i.e., immature B cells, naïve B cells, memory B cells, plasma cells, B1 and B2, germinal centre, or marginal zone B cells). 

In cord blood, the B-cell population is somewhat enriched as compared to adult peripheral blood. The mean frequency of B cells is about 11% as compared to approximately 5% in adult peripheral blood [1]. However, information regarding function and phenotype of B-cell subsets in cord blood is scanty. Recent studies by Griffin et al. [2] and Ichii et al. [3] have shown that human cord blood B cells may be divided into different subpopulations both phenotypically and functionally but more need to be done to fully understand early development of the B cells and their contribution in health and disease. 

It has been shown that CD5 is expressed on the first B cells detectable in the fetal liver and developing lymph nodes suggesting that CD5 may also be a marker of origin [4]. In cord blood, it has been shown that almost 60–80% of the B cells express CD5, and the expression decreases with age, finally ending up with a frequency of 5–30% in adult B cells [5, 6]. Furthermore, CD5 can be used for dividing mouse B cells into B1 and B2 B cells where B1a B cells express CD5 and B2 represent the conventional B cells [7–11]. The B1 B cells have been shown to produce low-affinity polyreactive immunoglobulins, both in mice and humans, recognising several autoantigens as well as many bacterial antigens (i.e., lipopolysaccharides and polysaccharides) [12–15]. Interestingly, CD5-expressing B cells have been suggested to follow separate distinct developmental pathways [16–18]. However, recent multiple studies have shown the expression of CD5 on other subsets of B cells than B1 B cells in humans indicating that a combination of markers including the CD5 is needed to correctly identify these cells in both humans and mice [19].

In adults, the B-cell production has switched from liver to be exclusively generated and matured in the bone marrow. The type 1 glycoprotein, CD27, is expressed on memory B cells and plasmablasts and can be found on approximately 40% of all circulating B cells in adults [20, 21]. In contrast, Agematsu et al. have shown that in cord blood the CD27-expressing population is found to be approximately 3% with increasing numbers with aging. 

We have previously described a subpopulation of immunoregulatory B cells based on the expression of the surface marker CD25 [22]. The CD25^+^ B cells isolated from peripheral blood have different phenotypical and functional properties as compared to CD25^−^ B cells [22]. The expression of CD25 is rapidly increased following toll-like-receptor-mediated cell stimulation, resulting in the expression of a fully functional IL-2R. This mechanism of IL-2R activation is nuclear factor-kappa *β* dependent. CD25^+^ B cells display a more mature and activated phenotype and belong to a subclass of memory B cells, based on analysis of immunoglobulins (Ig) expression, surface expression of the memory marker CD27, and the costimulatory molecule CD80 [22, 23]. Functionally, they seem to work as immunomodulatory B cells by better function as antigen-presenting cells and secreting the immunosuppressive cytokine IL-10, as compared to CD25^−^ B cells [22, 23]. In contrast, very few CD25^+^ B cells secrete immunoglobulins when compared to CD25^−^ B cells. Moreover, the proliferative response to IL-2 may suggest that CD25^+^ B cells express a fully functional high-affinity IL-2 receptor (IL-2R) [22, 23].

By use of freshly collected cord blood, we studied phenotype and function of CD25^+^ B cells as compared to CD25^+^ B cells isolated from adult peripheral blood, or to CD25^−^ B cells from cord blood. We found that only a few percentages of the cord blood B cells (CBC) expressed CD25. However, they expressed the complete high-affinity IL-2R, and the receptor was fully functional. A coexpression of higher frequency of CD5 on these cells may suggest a subset of B1 B cells in cord blood, but further more in depth studies are needed to evaluate this hypothesis. It seems like the CD25^+^ CBC already are designated to develop to memory B cells and are highly mobile due to a high expression of homing receptors. Finally, they spontaneously secrete immunoglobulins of the IgG and IgM subclass.

## 2. Material and Methods

### 2.1. Healthy Individuals

34 mothers giving birth at Mölndal Hospital in Göteborg, Sweden gave their informed consent to donate cord blood after delivery. From the cord, 10–50 mL blood was drawn into tubes containing heparin. Heparinized peripheral blood samples from 22 adult blood donors (age 30–65 years) was used as controls. This study was approved by the ethical committee at University of Göteborg, Göteborg, Sweden.

### 2.2. Cell Preparation

Peripheral blood mononuclear cells were isolated by density gradient centrifugation using lymphoprep (Axis-Shield, Oslo, Norway). For B-cell preparation cells were washed twice in phosphate buffered saline (PBS), the unspecific binding of antibodies by Fc-receptors was blocked with 5% fetal calf serum (FCS) for 5 minutes in room temperature (RT) followed by staining of cells surface CD20 (2H7 or L27, BD-Bioscience, Erembodegem, Belgium) and CD25 (2A3, BD-Bioscience) for 30 minutes in 4° and sorted using FACSAria (BD-Bioscience).

### 2.3. Phenotypic Analysis of B Cells

Phenotypic analysis of the cells was performed with a fluorescence-activated cell sorter (FACS) as described [22, 23]. The following mouse antihuman monoclonal antibodies (mAbs) were used: anti-CD5 (clone UCHT2), anti-CD20 (2H7 or L27), anti-CD25 (2A3), anti-CD27 (LI28), anti-CD80 (L307.4), anti-CD86 (2331), anti-CD122 (Mik-*β*3), anti-CD132 (Tugh4), and L-selectin (SK11), all purchased from BD, also CCR9 (112509), CCR7 (150503), CCR4 (205410), and CCR10 (314305), all purchased from R&D systems, Abingdon, UK. As isotype controls, mouse monoclonal IgG1 antibodies (clone X40 or PK136) directed against keyhole limpet hemocyanin were used. 

Surface immunoglobulin (IgA, IgD, IgG, and IgM) staining was performed using FITC-conjugated F(ab′)_2_ fragments of goat antihuman *γ*-, *μ*-, and *α*-specific antibodies (DakoCytomation, Glostrup, Denmark). As isotype controls, we used an irrelevant goat antihuman FITC-conjugated F(ab′)_2_ fragment also purchased from (DakoCytomation).

### 2.4. Proliferation

Sorted CD20^+^CD25^+^ or CD20^+^CD25^−^ B cells were stimulated with rh-IL-2 (25U) (R&D systems) and cultured at 2.5 × 10^5^ cells per well in RPMI medium containing 10% FCS, 1% gentamicin, 1% L-glutamine, and 1% mercaptoethanol, complete culture medium (all from Sigma-Aldrich) in round-bottomed ninety-six well plates at a final volume of 200 *μ*L. Cultured cells were incubated in a humidified atmosphere containing 5% CO_2_ at 37° and pulsed with [^3^H]-thymidine (Amersham Pharmacia Biotech, Little Chalfont, UK) for last 8 h of 96 h culture. The cells were harvested onto glass filters and dried, whereafter incorporated [^3^H]-thymidine was measured using a *β*-scintillation counter.

### 2.5. Cell Stimulation

2.5 × 10^4^ of CD20^+^CD25^+^ or CD20^+^CD25^−^ B cells per well were cultured in complete culture medium in round-bottomed ninety-six well plates at a final volume of 100 *μ*L. Cells were cultured in medium alone or stimulated either with 3 *μ*M backbone protected CpG-S 5′-TCGTCGTTTTGTCGTTTTGTCGTT-3′ (Scandinavian Gene Synthesis AB, Köping, Sweden), 5 *μ*g/mL Pam_3_Cys (EMC Microcollections, Tübingen, Germany), or 25U rIL-2 (R&D systems) in a humidified atmosphere containing 5% CO_2_ at 37°. Supernatants collected following 48 h and 72 h of stimulations were stored at −70° until used.

### 2.6. Cytometric Bead Array (CBA)

Cytokine (IL-2, IL-4, IL-6, IL-10, and IFN-*γ*) levels in supernatants were measured using the human Th1/Th2 cytokine CBA kit according to the manufacturers protocol (BD-Bioscience) and analysed using the FCAP array software (Soft Flow Inc, Minn, USA).

### 2.7. ELISPOT

Spontaneous secretion of antibodies from 2 × 10^4^ CD20^+^CD25^+^ and CD20^+^CD25^−^ B cell subsets was detected by the enzyme-linked immunosorbent spot (ELISPOT) technique as described in [22].

### 2.8. Statistical Evaluation

To calculate statistical differences, we used the parametric unpaired *t*-test when comparing results from cord blood versus results from adult blood, whereas when comparing results within each group, the paired *t*-test was used. *P ≤ 0.01, **P ≤ 0.001, and ***P ≤ 0.0001.

## 3. Results

### 3.1. IL-2R Expression on B Cells Isolated from Cord Blood as Compared to Adult Peripheral B Cells

**Figure 1:**
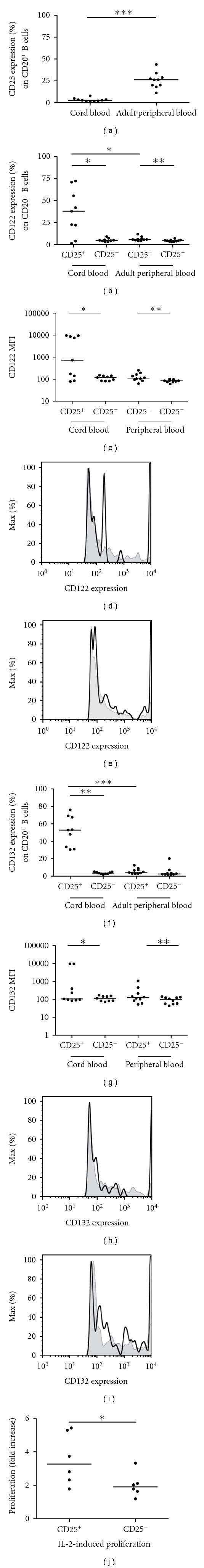
Expression and functional evaluation of the IL-2R on cord blood and adult CD20^+^ B cells. From freshly isolated cord blood (*N* = 10) or adult blood, mononuclear cells (*N* = 10) were isolated by density gradient centrifugation. Cells were stained for 3-colour flow cytometry analysis using a combination of CD20, CD25, CD122, or CD132. (a) shows the CD25 expression on CD20^+^ cells isolated from cord blood or adult blood. In (b), the CD122 expression and in (f) the CD132 expression are analysed on CD25^+^ and CD25^−^ CD20^+^ B cells isolated from cord blood or adult blood. In (c) the median intensity of CD122 and in (g) the median intensity of CD132 are shown on CD25^+^ and CD25^−^ CD20^+^ B cells isolated from cord blood or adult blood. (d) and (h) show a representative histogram of CD122 (d) and CD132 expression on CD25^+^ and CD25^−^ cord blood B cells. Black line indicates CD25^+^ and filled histogram represent CD25^−^ cord blood B cells. (e) and (i) show a representative histogram of CD122 (d) and CD132 expression on CD25^+^ and CD25^−^ adult B cells. Black line indicates CD25^+^ and filled histogram represent CD25^−^ adult B cells. Finally, in (j), the proliferation of ^3^H-thymidine labelled sorted CD25^+^ and CD25^−^CD20^+^ B cells isolated from cord blood (*N* = 6) is shown following 96 h of stimulation with 25U rh-IL-2. Line in boxes represents median. Statistical evaluation was performed using the parametric unpaired *t*-test when comparing results from cord blood versus results from adult blood, whereas when comparing results within each group, the paired *t*-test was used. *P ≤ 0.01, **P ≤ 0.001, and ***P ≤ 0.0001.

When analysing the surface expression of the IL-2R alpha subunit CD25 on B cells isolated from human umbilical cord blood, we found a significantly lower frequency (approximately 5%) as compared to B cells isolated from adult peripheral blood (approximately 25%) (*P* ≥ 0.0001) (Figure 1(a)). Interestingly, when dividing the CBC into CD25^+^ and CD25^−^ cells, analysing the expression of the other two subunits of the IL-2R (CD122 and CD132) CD25^+^ CBC expressed significantly higher frequencies as compared to adult corresponding cells (*P* ≥ 0.002 and *P* ≥ 0.0001, resp.) (Figures 1(a) and 1(f)).

Comparing CD25^+^ and CD25^−^ CBC regarding the expression of CD122 and CD132, the CD25^+^ population displayed significantly higher frequencies (*P* ≥ 0.006, *P* ≥ 0.0001) (Figures 1(b) and 1(f)). In contrast, the adult CD25-expressing B-cells only displayed a higher frequency of CD122 as compared to the CD25^−^ B-cell population (*P* ≥ 0.02) (Figure 1(f)).

When analysing the median fluorescence intensity of the CD122 and CD132 expression on CD25^+^ and CD25^−^ from CBC and adult blood, we found an increased intensity of CD122 but a decreased intensity of CD132 on CD25^+^ B cells from CBC (*P* ≥ 0.03 and *P* ≥ 0.04) (Figures 1(c) and 1(f)). On adult CD25^+^ B cells, the intensity of both CD132 and CD132 was increased (*P* ≥ 0.01, *P* ≥ 0.002) (Figures 1(c) and 1(g)).

### 3.2. CD25^+^ CBC Express a Fully Functional IL-2 Receptor

To investigate if CD25^+^ CBC not only expressed the high-affinity IL-2 receptor but also if it was fully functional, we stimulated the CBC subpopulations with rh-IL-2. We found that CD25^+^ CBC proliferated significantly more in response to rh-IL2 as compared to CD25^−^ CBC (*P* ≥ 0.04) (Figure 1(j)). To role out that the detected increased proliferation of the CD25^+^ CBC following rh-IL-2 stimulation was dependent on an increased CD25 expression, we also analysed the CD25 expression on CBC following 24 h of rh-IL-2 addition. No increased CD25 expression could be detected on CBC following 24 h of rh-IL-2 stimulation (data not shown).

### 3.3. CD25^+^CBC Express Both CD5 and the Human B-Cell Memory Marker CD27

**Figure 2:**
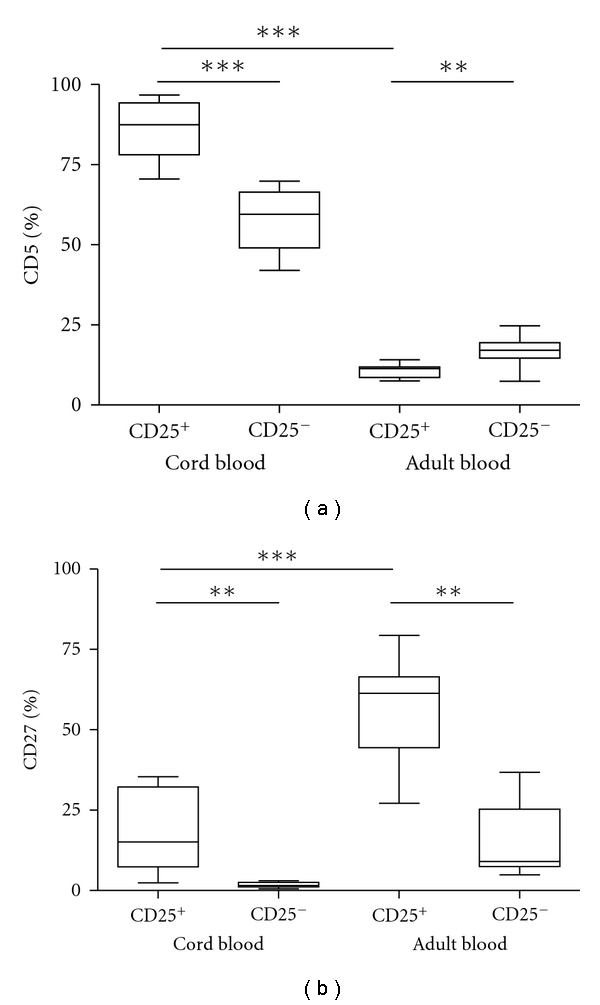
Expression of the CD5 and CD27 epitopes in CD25^+^ and CD25^−^ CD20^+^ B cells isolated from cord blood and adult blood. From freshly isolated cord blood (*N* = 10) or adult blood, mononuclear cells (*N* = 10) were isolated by density gradient centrifugation. Cells were stained for 3-colour flow cytometry analysis using a combination of CD20, CD25, CD5, or CD27. In (a) the CD5 and in (b) the CD27 expression on CD25^+^ and CD25^−^ CD20^+^, B cells isolated from cord blood or adult blood are shown. Line in boxes represents median. Statistical evaluation was performed using the parametric unpaired *t*-test when comparing results from cord blood versus results from adult blood, whereas when comparing results within each group, the paired *t*-test was used. *P ≤ 0.01, **P ≤ 0.001, ***P ≤ 0.0001.

To further determine the maturation and memory status on CD25^+^ CBC, we examined the surface expression of CD5 and CD27 on these cells. We found that CD25^+^ CBC expressed a significantly higher frequency of CD5 as compared to the corresponding population of ABC (*P* ≥ 0.0001) (Figure 2(a)). On CD25^+^ CBC, almost 85% expressed CD5 as compared to 55% of the CD25^−^ CBC (*P* ≥ 0.0001) (Figure 2(a)). In sharp contrast, only 10% of the adult CD25^+^ B cells expressed CD5, and a significantly increased frequency was found on the CD25^−^ B-cell population (*P* ≥ 0.0049) (Figure 2(a)).

Analysing the surface expression of the human memory marker CD27 on CD25^+^ CBC, we found a significantly lower frequency of CD27 than the corresponding ABC (*P* ≥ 0.0001) (Figure 2(b)). 

Comparing CD25^+^ CBC to CD25^−^CBC, a significantly higher frequency (*P* ≥ 0.0019) of CD27 was detected on the CD25^+^ CBC (Figure 2(b)). The same result was found on the CD25^+^ ABC compared to CD25^−^ B cells (*P* ≥ 0.0001) (Figure 2(b)).

### 3.4. CD25^+^ CBC Display Similar Immunoglobulin Expression Compared to CD25^+^ ABC

When comparing CD25^+^ CBC to CD25^+^ ABC regarding the surface expression of the immunoglobulins A, D, G, and M, we found no statistical significant differences between the two groups, even though adult CD25^+^ B cells displayed a clear trend of more IgA and IgG expressing cells. 

**Figure 3:**
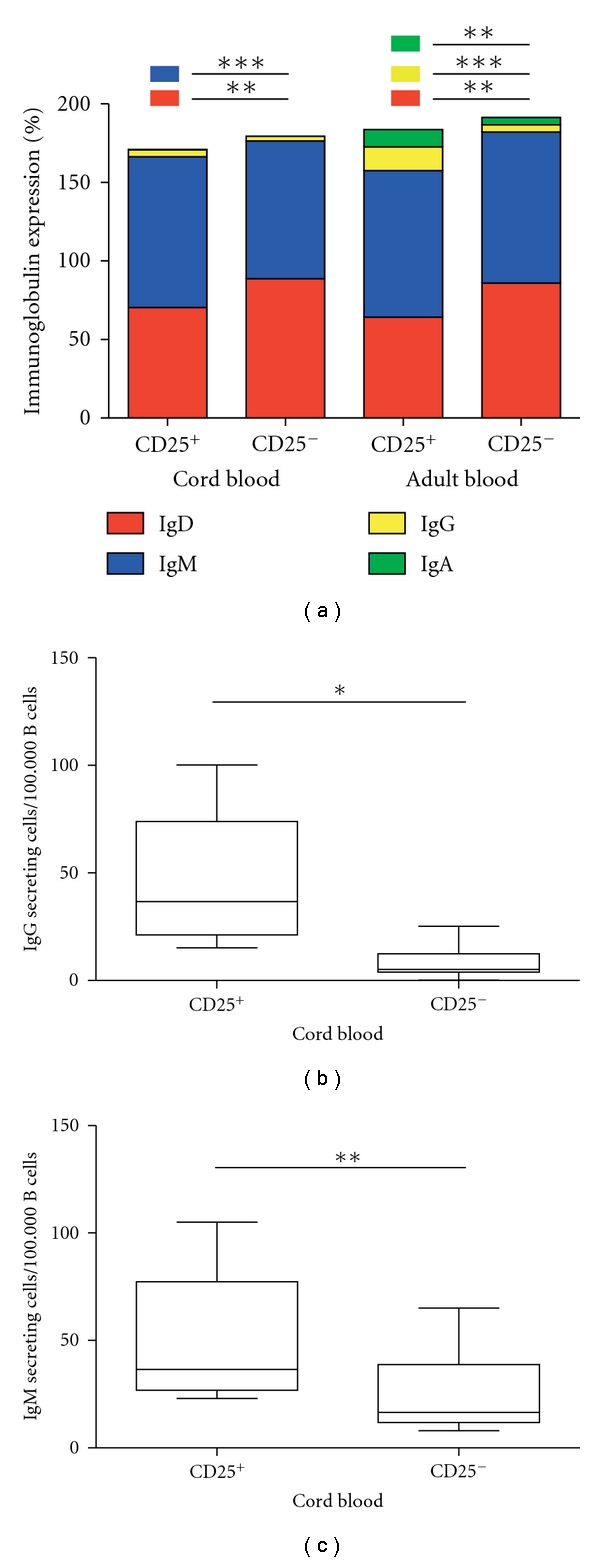
Analysis of surface immunoglobulin expression and secretion from CD25^+^ and CD25^−^ CD20^+^ B cells isolated from cord blood and adult blood. In (a) freshly isolated cord blood (*N* = 10) or adult blood, mononuclear cells (*N* = 10) were isolated by density gradient centrifugation. Cells were stained for 3-colour flow cytometry analysis using a combination of CD20, CD25, IgG, IgA, IgM, and IgD. In (a), a summary of all 4 surface immunoglobulins expressed by the CD20^+^CD25^+^ or CD20^+^CD25^−^ B cells is shown. Red colour represents IgD, blue colour represents IgM, yellow colour represents IgG, and green colour represents IgA. In (b) and (c), freshly isolated cord blood mononuclear cells (*N* = 6) were isolated by density gradient centrifugation followed by flow cytometry sorting into CD20^+^CD25^+^ or CD20^+^CD25^−^ populations. These populations were then analysed for the capacity to secret either IgG (b) or IgM (c) by use of ELISPOT. Line in boxes represents median. Statistical evaluation was performed using the parametric unpaired *t*-test when comparing results from cord blood versus results from adult blood, whereas when comparing results within each group, the paired *t*-test was used. *P ≤ 0.01, **P ≤ 0.001, and ***P ≤ 0.0001.

CD25^+^ CBC expressed significantly increased frequencies of IgM (*P* ≥ 0.0001) but a decreased frequency of IgD (*P* ≥ 0.0061) as compared to CD25^−^ CBC (Figure 3(a)). The surface expression of IgG and IgA on CBC was very low, and accordingly, no differences in the frequency of IgG and IgA expression were found between the two groups. 

In contrast, CD25^+^ ABC displayed a significantly higher frequency of IgG (*P* ≥ 0.0001) and IgA (*P* ≥ 0.0007), but a lower IgD (*P* ≥ 0.0005) expression as compared to the CD25^−^ B cells (Figure 3(a)).

### 3.5. CD25^+^ CBC Spontaneously Secrete IgG and IgM *In Vitro*

Sorted CD25^+^ CBC were compared to CD25^−^ CBC regarding their ability to secrete IgM, IgG, and IgA using ELISPOT. Interestingly, we found a significantly increased numbers of IgG, and IgM secreting CD25^+^ CBC as compared to CD25^−^ CBC (*P* ≥ 0.05 for both) (Figures 3(b) and 3(c)). No or only a few cells secreted IgA, and no differences were detected between the populations (data not shown).

### 3.6. CD25^+^ CBC Display High Frequencies of Homing Receptors

To investigate the capacity of migration to different tissues in the body, we examined the expression of selected homing receptors (i.e., CCR4, CCR7, CCR9, CCR10, and L-selectin) on CD25^+^ and CD25^−^ CBC and compared them to each other or to corresponding ABC. We found that CD25^+^ CBC expressed CCR4, CCR7, and CCR9 when compared to the corresponding population in adults, this was a significantly increased expression (Table 1). In contrast, CD25^+^ CBC expressed significantly decreased frequencies of L-selectin as compared to CD25^+^ B cells from adults (Table 1). 

Furthermore, the frequency of the CCR4, CCR7, CCR9, CCR10, and L-selectin expression was significantly increased on CD25^+^ as compared to CD25^−^ CBC (Table 1). 

Analysing CD25^+^ ABC, we found that frequency of CCR4, CCR7, CCR9, CCR10, and L-selectin was significantly increased as compared to CD25^−^ B cells (Table 1).

### 3.7. Expression of the Costimulatory Molecules CD80 and CD86 on CD25^+^ CBC as Compared to CD25^+^ ABC

When comparing the costimulatory molecule expression of CD80 and CD86 on CD25^+^ B cells from CBC to the corresponding ABC population, we found that the CD80 frequency was significantly decreased on the CD25^+^ CBC (Table 1). In contrast, the frequency of CD86 was significantly increased on the CD25^+^ CBC as compared to the CD25^+^ ABC (Table 1).

Furthermore, CD25^+^ CBC displayed a significantly increased frequency of CD80 and CD86 as compared to CD25^−^ B cells (Table 1). On CD25^+^ ABC, only CD80 expression was significantly increased as compared to CD25^−^ B cells (Table 1). 

### 3.8. Cytokine Production from CD25^+^ B Cells from CBC

Following addition with selected stimulus for 48 or 72 hours, analysis of cytokine secretion from CD25^+^ and CD25^−^ CBC into the supernatants was performed. No significant differences could be detected for any of the investigated cytokines (i.e., IL-2, IL-4, IL-6, IL-10, or INF-*γ*) (data not shown).

## 4. Discussion

In the present study, we have investigated CD25-expressing B cells from the human cord blood. We have been able to show that cord blood B cells express significantly lower frequency of CD25 when compared to adult B cells that express CD25 in about 30%. Further, we have found that the CD25^+^ B cells from cord blood expressed a fully functional high-affinity IL-2 receptor, proven by the significantly increased proliferation seen following stimulation with rh-IL-2 *in vitro*. We have previously shown that adult CD25^+^ B cells proliferate in response to IL-2 [22]. Using our new results, we can see that CD25^+^ CBC have the capacity to proliferate in the same extent as adult peripheral cells. This suggests that CD25^+^ CBC have the ability to respond and multiply in situations where IL-2 levels are increased.

Furthermore, cord blood CD25^+^ B cells also expressed increased frequencies of CD122 and CD132. We found increased intensity of CD122 but decreased intensity of CD132 on cord blood CD25^+^ B cells. Since CD122 is part of the IL-15R, and the common gamma chain, CD132, is part of IL-4R, IL-7R, and IL-9R, these cells may respond to other cytokines too. Many of these cytokines are involved in early maturation processes of B cells [24].

We continued our investigation by analysing the CD5 expression on these cells. It has been postulated that CD5 is expressed on less mature B cells and is known to be downregulated when the B cells mature with age [5, 6, 25]. In mice, CD5 expression is associated with the B1a B cell subclass [7–11]. A subclass that secretes low-affinity IgM antibodies usually is directed against bacterial cell wall components [12–15]. However, in humans, this B1a population has not been clearly verified, published data suggests that similar subclasses of B cells may exist, and, in fact, it has been suggested that CD5-expressing B-cell subsets are involved in the production of autoreactive antibodies [12–15] and reviewed in [26]. Recent publication describing a human B1 subset of B cells has however shown that these cells may be CD5 negative but express CD27 and CD43 [2], We found a clear distinction between cord blood CD25^+^ and adult blood CD25^+^ B cells regarding the expression of the CD5 and memory CD27 epitopes. Even though the discussion regarding B1 B cells in humans is still ongoing, we may speculate that CD25-expressing B cells may be part of a subset belonging to B1 cells, at least in cord blood and during the early life.

Antibody production is one of the main functions of the B cells. We were able to show that cord blood CD25^+^ B cells have the ability to spontaneously secrete antibodies. This secretion was not as efficient as the adult counterpart as we previously have shown [22]. Though, we did not analyze the specificity of these antibodies, we believe our results are of interest as they show that CD20^+^CD25^+^ B cells in cord blood coexpressing CD5 and CD27 are not just activated B cells but have the ability to produce antibodies of IgM and IgG isotype. These results need further investigation to fully understand the origin and function of these cells. 

B cells have been shown to produce a line of various cytokines [27, 28], In the last decade, the upcoming subset of regulatory B cells with the capacity to produce IL-10 and downregulate inflammation in a line of animal disease models has further indicated the importance of cytokine production by B cells [29, 30]. In our previous study, we show that CD25 + B cells from peripheral adult blood do produce IL-10 after TLR stimulation [23]. Here, we did not see any difference when comparing two subsets of the B cells in cord blood regarding the cytokine production. However, we cannot conclude that these cells do not produce cytokines in low levels after specific stimulation or do need to be activated in the presence of other immunological cell types in order to produce them. 

To further characterise the CD25^+^ CBC, we choose to investigate the CC-chemokine receptor expression on these cells. We found that the expression of homing receptors was increased in both CD25^+^ B cells isolated from cord blood and from adult peripheral blood as compared to CD25^−^ B cells. Homings receptors are expressed by various immune cells in order to migrate to the sight where they will be needed. We found higher expression of CCR7, involved in B-cell homing to secondary lymphoid tissue and T-cell zone [31] and higher expression of CCR9 and CCR10 both homing receptors involved in homing to intestinal tissue [32, 33] on CD25^+^ B cells in cord blood. This is just a phenotypical analysis but may be an indication for the function or upcoming function of these cells. Interestingly, when CD25^+^ B cells are isolated from adult peripheral blood, the homing receptor expression seems to be downregulated as compared to CD25^+^ CBC. This might suggest that CD25^+^ B cells during fetal life may be of importance for colonising the peripheral immune organs. 

Conclusively, we found that cord blood CD25^+^ B cells share some characteristics to adult corresponding cells that is expression of a fully functional IL-2 receptor and increased expression of activation and memory markers. The similarities in homing receptor expression also suggest a highly mobile phenotype, and the capacity of CD25^+^ CBC to spontaneously secrete immunoglobulins suggests a protective role and important properties in the immune regulation early in life. 

## Figures and Tables

**Table 1:** Comparison of homing receptor and activation marker expression between CD20^+^CD25^+^ or CD20^+^CD25^−^ cells isolated from human cord blood or from adult peripheral blood.

	Cord blood		Adult peripheral blood		*P* value
	CD20^+^CD25^+^	CD20^+^CD25^−^	CD20^+^CD25^+^	CD20^+^CD25^−^	
CCR4	35.7 ± 5.5	3.0 ± 0.93	10.8 ± 0.77	6.4 ± 0.85	0.0006^†^
					0.0001^‡^
					0.001^§^
CCR7	94.1 ± 1.4	68.3 ± 3.1	84.5 ± 2.7	76.1 ± 3.7	0.0001
					0.003
					0.006
CCR9	20.0 ± 3.5	3.9 ± 0.9	10.6 ± 1.4	8.2 ± 1.5	0.002
					0.005
					0.03
CCR10	17.5 ± 4.6	3.1 ± 2.6	16.5 ± 1.5	12.9 ± 1.4	0.003
					0.001
					n.s
L-selectin	87.4 ± 3.5	73.6 ± 6.9	97.8 ± 0.7	94.8 ± 0.8	0.006
					0.003
					0.04
CD80	3.8 ± 3.3	0.68 ± 0.08	21.9 ± 2.5	8.8 ± 1.9	0.04
					0.0006
					0.03
CD86	10.3 ± 3.8	2.5 ± 0.37	0.63 ± 0.32	0.63 ± 0.23	0.02
					n.s
					0.05

Values are expressed as frequency of expression in %  ± SEM.

^†^Comparison between cord blood CD20^+^CD25^+^ and CD20^+^CD25^−^.

^‡^Comparison between peripheral adult blood CD20^+^CD25^+^ and CD20^+^CD25^−^.

^§^Comparison between cord blood CD20^+^CD25^+^ and peripheral adult blood CD20^+^CD25^−^.

## Abbreviations

ABC: Adult blood B cells
CBC: Cord blood B cells
CD: Cluster of differentiation
ELISPOT: Enzyme-linked immunosorbent spot
FACS: Fluorescence-activated cell sorter
FCS: Fetal calf serum
IG: Immunoglobulin
IL: Interleukin
mABS: Monoclonal antibodies
PBS: Phosphate buffered saline.
